# Gastric Desmoid Fibromatosis – Report of a Rare Mimic of Gastrointestinal Stromal Tumor

**DOI:** 10.7759/cureus.19614

**Published:** 2021-11-15

**Authors:** Jim C Lee, David Curtis, Jonathan B Williamson, Saverio Ligato

**Affiliations:** 1 Pathology, Hartford Hospital, Hartford, USA; 2 Surgery, Hartford Hospital, Hartford, USA; 3 Medicine, Hartford Hospital, Hartford, USA

**Keywords:** endoscopic us-guided fine-needle aspiration and biopsy, gastric submucosal spindle cell lesions, intra-abdominal fibromatosis, gastric desmoid tumor, desmoid fibromatosis

## Abstract

Desmoid fibromatosis (DF) involving the gastrointestinal tract is extremely rare. Its intramural location and occasional expansile growth pattern within the bowel wall may mimic a gastrointestinal stromal tumor (GIST). Due to the different disease behaviors and management, it is important to make a correct diagnosis before further treatment. We present an extremely rare case of a gastric DF that on imaging appeared as a discrete intramural mass mimicking a GIST and that was preoperatively correctly diagnosed as a DF based on its cytomorphologic, immunohistochemical, and molecular profiles.

The patient is a 71-year-old female who presented with dysphagia and unintentional weight loss. A mass was identified at the gastric fundus. Endoscopic ultrasound-guided fine-needle aspirate (FNA) and biopsy (FNB) were performed. The FNA showed a few small aggregates of cytologically bland spindle-shaped cells with elongated nuclei. The FNB yielded small fragments of tissue composed of bland spindle cells demonstrating nuclear and cytoplasmic immunostain for β-catenin and focal stain for smooth muscle actin (SMA) and desmin. CD117, DOG1, CD34, caldesmon, S100, cytokeratin AE1/AE3, signal transducer and activator of transcription 6 (STAT6), MUC4, progesterone receptor (PR), and anaplastic lymphoma kinase (ALK) were negative, and MIB-1 showed a very low proliferation activity index. Molecular studies performed by targeted next-generation sequencing showed activating mutations in CTNNB1*.* These results excluded a GIST and confirmed the diagnosis of a gastric DF.

Although it is very rare, DF must be included in the differential diagnosis of discrete intramural gastric spindle cell lesions. A definitive diagnosis can be made preoperatively if enough lesional material is available for appropriate immunohistochemical and molecular studies.

## Introduction

Desmoid fibromatosis (DF), also known as desmoid tumor and aggressive fibromatosis, is a locally aggressive non-metastasizing myofibroblastic neoplasm with an infiltrative growth pattern and propensity for local recurrence [1]. The most commonly reported locations of extra-abdominal DF are the abdominal wall (50%) and the extra-abdominal soft tissues in the trunk or limbs (40%). Intra-abdominal fibromatosis is the least common (8 %) and affects primarily the mesentery of the small bowel, the ileocolonic region, and the mesocolon. Sometimes it may also encroach on, extend into, or even arise from the intestinal wall mimicking a gastrointestinal stromal tumor (GIST) [2]. Here we present a rare case of an intramural gastric DF mimicking a GIST.

## Case presentation

**Figure 1:**
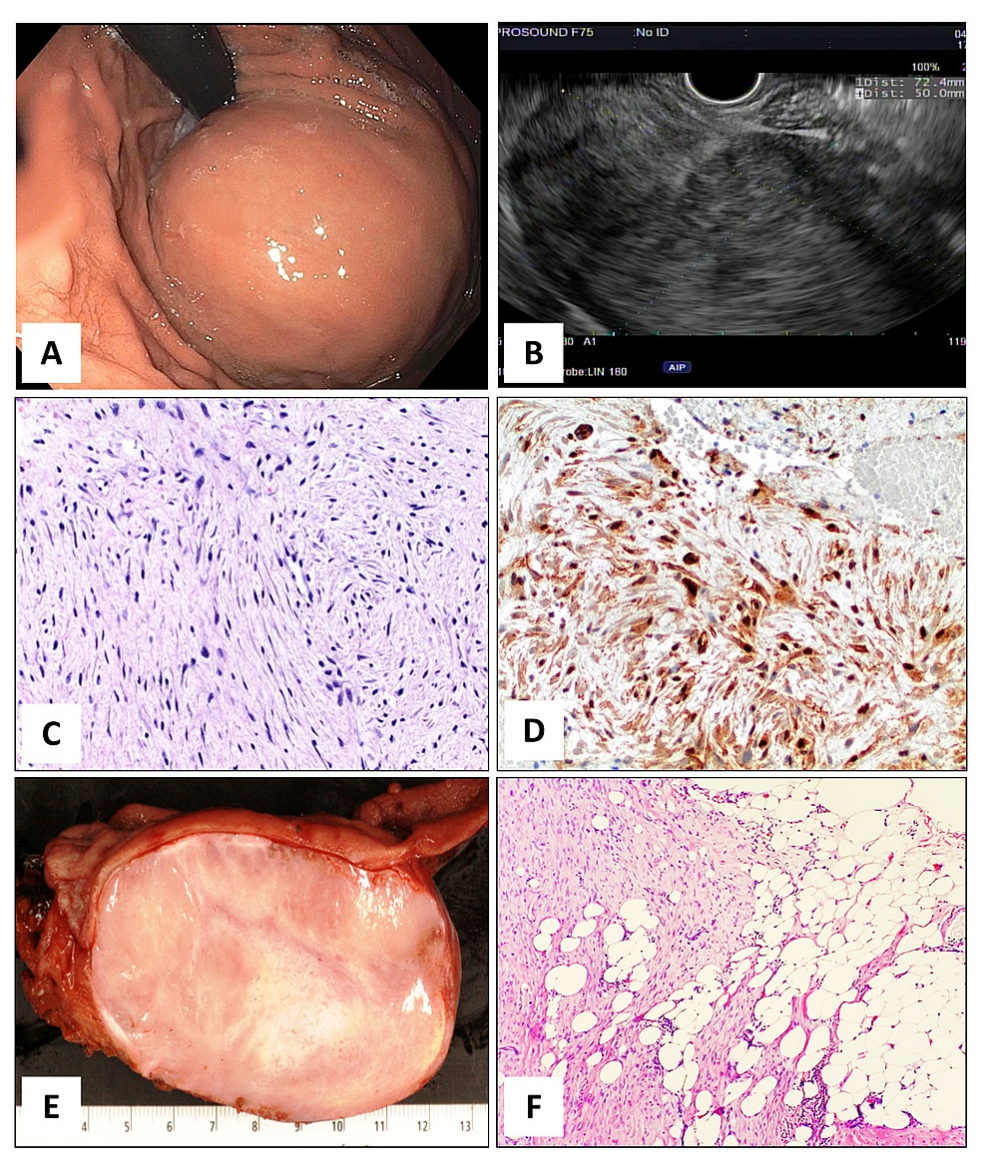
Macro and microscopic features Figure 1A: Endoscopic picture of intact gastric mucosa with bulging subepithelial mass in the cardia/fundus of the stomach visible on retroflexion view. Figure 1B: Endoscopic ultrasound image reveals a hypoechoic well-defined gastric mass deriving from the muscle wall Figure 1C: FNB of the gastric lesion with long sweeping fascicles of elongated and slender spindle cells. (40X, H&E) Figure 1D: Immunohistochemical stain for β-Catenin shows strong and diffuse nuclear and cytoplasmic labeling in the tumor cells. (40X, IHC) Figure 1E: Cross-section of the resected gastric mass reveals a well-demarcated intramural heterogenous mass with discrete contours. The overlying mucosa (upper portion of the picture) and the serosal surface (lower portion of the picture) are both intact. The esophageal-gastric junction is on the left side of the picture. Figure 1F: Focally the tumor infiltrates into the subserosal gastro-epiploic fat. (10X, H&E)

A 71-year-old female presented with dysphagia, intermittent left upper abdominal pain, and unintentional weight loss of approximately seven lbs during the last four months. Her past medical history was significant for Barrett’s esophagus, laparoscopic left salpingo-oophorectomy for an ovarian cystadenoma, and repair of aortic stenosis with aortic valve replacement. Physical examination revealed left upper abdominal tenderness on deep palpation. An abdominal CT scan with contrast demonstrated an 8.5 x 6.0 x 5.5 cm solid, well-marginated mass along the fundus of the stomach, extending to the gastroesophageal junction with inhomogeneous contrast enhancement, and it was thought to be most consistent with a GIST. Upper gastrointestinal endoscopy showed a large subepithelial gastric lesion with no bleeding or ulceration of the mucosa located in the cardia/fundus of the stomach (Figure 1A). Endoscopic ultrasound (EUS) revealed a hypoechoic subepithelial gastric lesion in the cardio-fundic region measuring 7.2cm x 5cm which appeared to originate from the muscular wall of the stomach. The outer endosonographic borders were well defined, and an intact interface was noted between the mass and the adjacent structures, suggesting a lack of invasion (Figure 1B).

Fine needle aspiration (FNA) and biopsy (FNB) were performed. The FNA showed very scant cellularity with a few small aggregates of cytologically bland spindle-shaped cells with elongated nuclei in a myxoid background. The FNB yielded small fragments of tissue composed of bland spindle cells in a fibro-myxoid stromal background containing small elongated blood vessels, and no mitosis or necrosis was identified (Figure 1C). The differential diagnosis included a GIST, leiomyoma, schwannoma, inflammatory myofibroblastic tumor, and other rare mesenchymal neoplasms, including a plexiform fibromyxoma. Immunohistochemical stains, performed in the small fragments of tissue obtained by FNB, were negative CD117, DOG1, CD34, caldesmon, S100, cytokeratin AE1/AE3, signal transducer and activator of transcription 6 (STAT6), and anaplastic lymphoma kinase (ALK). MIB-1 showed a very low proliferation index (~3%). Instead, a strong nuclear and cytoplasmic immunoreactivity for β-catenin (Figure 1D) was identified in the spindled cells, along with focal cytoplasmic positivity for smooth muscle actin (SMA). These results raised the possibility of a rare case of a primary gastric DF. Molecular analysis performed by targeted next-generation sequencing (NGS) revealed activating mutations in the CTNNB1 gene. These results confirmed the diagnosis of a gastric DF, most likely sporadic type, and definitively excluded the possibility of a GIST.

The patient underwent a proximal gastrectomy for curative treatment. A well-demarcated bulging intramural ovoidal mass measuring 9.2 cm x 7.5 cm x 5.5 cm abutting the serosa was removed with negative resection margins. On gross examination, the gastric mucosa was freely movable and flattened without bleeding or ulcerations. The cut surface of the mass showed an ovoid lesion with discrete contours and heterogeneous, tan-white fibrous central areas alternating with more myxoid peripheral areas. No gross evidence of infiltration into the adjacent structures was identified (Figure 1E).

Microscopically, the cellularity of the lesion was quite heterogeneous, with sections taken from the central portion of the tumor, demonstrating uniform, cytologically bland, spindle cells with long sweeping fascicles in a fibrotic background alternating with more cellular areas showing a storiform pattern. Sections from the peripheral areas were instead less cellular and demonstrated a more myxoid stromal background in which irregular stag-horn thin-walled blood vessels could be identified.

Despite extensive sampling of the lesion, only two foci of early infiltration (5% of the lesion) into the subserosal gastro-epiploic fat were identified (Figure 1F). A final diagnosis of gastric DF was rendered and the patient recovered well and was discharged on postoperative day five. Currently, after 18 months post-surgery, she is well and without evidence of disease.

## Discussion

Gastric DF presenting as a discrete intramural mass with an expansile growth pattern mimicking a GIST is extremely rare [3,4]. However, this distinction has become clinically and therapeutically very important. Unlike GISTs, DF is not responsive to Imatinib-mesylate (Novartis, Basel, Switzerland) and does not have the capacity to metastasize, although it may recur locally. One of the most interesting aspects of this report is that when the case was presented at the gastro-intestinal multidisciplinary team, the clinical and imaging studies were highly suggestive of a large gastric GIST. The option of pre-operative treatment with Imatinib-mesylate was considered. However, the FNA and FNB demonstrated a spindle cell proliferation with an immunohistochemical and molecular profile supporting the diagnosis of DF and excluding a GIST. Hence, only surgery was performed. This case exquisitely demonstrates the value of the histopathological examination in helping the multidisciplinary team to identify the correct diagnosis and proceed with the appropriate surgical treatment.

Searching the literature, we found nine cases of primary gastric DF (Table 1) [3-10]. They occur most commonly between the third and fourth decade of life (age range: nine to 71 years old), almost equally affecting both sexes (4M and 5F) and ranging in size from 4 to 19 cm. The symptomatology is related more to the location of the lesion than its size. In fact, patients with DF localized in the gastric antrum presented with abdominal pain and vomiting, whereas those with tumors localized in the proximity of the gastroesophageal junction reported abdominal pain and dysphagia.

**Table 1:** Summary of nine cases of gastric desmoid fibromatosis NA: not available; ANED: alive with no evidence of disease; AWD: alive with disease; GE: gastroesophageal; EMA: epithelial membrane antigen; SMA: smooth muscle actin; F/U: follow-up; *: current case

No.	Sex	Age	Symptoms	Tumor location/ gross description	Tumor size (mm)	Ultrasonography and endoscopy findings	Surgical resection	Adjuvant therapy	Immunohistochemistry	Recurrence	Follow-up
1 [5]	F	15	NA	GE- junction	NA	NA	NA	NA	NA	NA	NA
2 [6]	M	67	NA	Posterior wall of stomach	NA	NA	NA	Imatinib for coexisting GIST	NA	NA	AWD (45 months F/U)
3 [7]	M	9	Abdominal pain, vomiting, weight loss	GE-junction	NA	Hypoechoic mass endoscopy did not pass the cardia.	R0	No	Positive: SMA, vimentin negative: S100, CD34, desmin	No	ANED (12 months F/U)
4 [8]	M	56	NA	Remaining greater curvature of the stomach, prior distal gastrectomy	40 x 40	Hypoechoic mass with clear boundaries Submucosal tumor.	R0/R1	NA	Positive: β-catenin negative: NA	No	ANED 12 months F/U)
5 [9]	F	37	Abdominal pain, vomiting	Antrum/ well-defined mass	190 x 150	Compression of gastric antral mucosa.	R0	No	Positive: β-catenin negative: CD117, CD34, S100, DOG1, Actin, bcl-2	No	ANED (48 months F/U)
6 [3]	F	47	Upper abdominal pain	Posterior wall of antrum	45 x 40 x 35	Hypoechoic mass with less clear boundaries. Ulcerated mucosa.	R1	No	Positive: SMA, β-catenin negative: CD117, CD34, S100	No	ANED (63 months F/U)
7 [4]	M	47	Abdominal pain	Antrum/ ill-defined boarders	58 x 43 x 34	Swelling of antral mucosa	R0	No	Positive: β-catenin, SMA negative: S100, desmin, CD34, CD117	No	ANED (13 months F/U)
8 [10]	F	45	Asymptomatic	Posterior gastric wall with extension into pancreas	55x45x40	Hypoechoic mass extending from the gastric muscular wall to the pancreatic tail. Bulging submucosal mass in the posterior wall of the middle gastric body.	R0	No	Positive: β-catenin, vimentin, SMA negative: Cytokeratin, EMA, S100, desmin, CD99, bcl-2, ALK, CD34, CD68, CD163, CD21, CD23, CD117, DOG1	No	NA
9*	F	71	Dysphagia, abdominal pain, weight loss	GE-junction/ fundus	92 x 75 x 55	Hypoechoic mass with well-defined boarders. Submucosal bulging tumor.	R0	No	Positive: β-catenin, CD10, SMA negative: CD117, DOG1, CD34, desmin, ALK, S100, cytokeratin AE1/AE3, STAT6, MUC4, PR.	No	ANED (4 months F/U)

Macroscopically, gastric DFs appear as large masses preferentially located either at the gastroesophageal junction or near the antrum. The cut surface shows a whorled, firm, tan-gray fibrous parenchyma without hemorrhage, necrosis, or cyst formation. They originate from the muscle layer and usually infiltrate all layers of the stomach with occasional ulceration of the mucosa or extension into the adjacent organs. However, occasionally, as in our case, DF may present as a well-circumscribed mass resembling a gastric GIST.

Microscopically gastric DFs consist of long sweeping fascicles of a slender spindle or stellate cells with tapering ovoid nuclei and inconspicuous or small nucleoli. No nuclear hyperchromasia or cytological atypia is usually identified. The vasculature is variably prominent, composed of thick blood vessels or thin, delicate blood vessels with a stag-horn pattern. Although in some cases the gross appearance of gastric DFs may appear circumscribed, microscopically, usually they show an infiltrating pattern of growth within the adjacent gastric muscle layer, into the serous layer, adjacent gastro-epiploic fat, and sometimes into the overlying mucosa. In this regard, our case is unique due to its near-total macroscopic and microscopic expansile growth pattern characterized by a spindle cells proliferation pushing aside the adjacent structures. In fact, the gastric mucosa, submucosa, and muscularis propria were not infiltrated by the neoplastic cells, and a thin layer of dense collagenous tissue was identified between the tumor and the adjacent muscularis propria. Although the great majority of our tumor showed pushing borders, two foci in which the spindle cells showed early infiltration into the adjacent serosal gastro-epiploic fat (Figure 1F).

The immunohistochemistry profile of DF is characterized by diffuse nuclear labeling for β-catenin and variable positivity for desmin and SMA. Dysregulation of the WNT/beta-catenin signaling pathway is identified in all tumors, and it is due either to somatic activating mutations in the CTNNB1 gene or germline inactivating mutations in the adenomatous polyposis coli (APC) gene [1]. Approximately 85-95% of sporadic DFs are characterized by CTNNB1 activating mutations in exon 3 [11]. Abnormalities affecting the genes governing tissue repair have been suggested to be the underlying cause of DF development. It is assumed that DF develops when stimulating factors are added to these genetic abnormalities. Stimulating factors include APC gene abnormalities such as familial adenomatous polyposis (FAP) and Gardner syndrome, mechanical stimulation such as laparotomy and abdominal injury, and changes in estrogen receptors during pregnancy and after delivery [12]. In our case, the prior laparotomy may be the stimulation of the DF; however, the proximity of the surgical and the lesional sites is unavailable. 

The clinical behavior of DF is unpredictable, with the sporadic type DF reportedly having a relatively better prognosis than those associated with FAP. The mortality rate for all cases with intra-abdominal fibromatosis is approximately 30%, and spontaneous regression has been reported in abdominal wall lesions but rarely in intra-abdominal sites. Recurrence is usually associated with young age and larger tumor size [1,2,12]. The standard of care for DF is surgical resection with clear margins.

The main differential diagnosis should be made with GIST, inflammatory myofibroblastic tumor, inflammatory fibroid polyp, leiomyoma, schwannoma, solitary fibrous tumor, and plexiform fibromyxoma.

## Conclusions

In summary, the value of this short report is to raise awareness among surgeons and pathologists that, although rare, primary gastric DF should be included in the differential diagnosis of gastric intramural spindle cells mesenchymal neoplasms to avoid a misdiagnosis of a GIST or other spindle cell lesions.
